# Barriers faced by undergraduate dental students when conducting research: a qualitative study

**DOI:** 10.1186/s12909-025-06790-y

**Published:** 2025-02-06

**Authors:** Mohammed Mahmoud Sarhan, Lama Nasser Aljohani, Raghad Ibrahim Alsaleh, Roze Abdulaziz Mubaraki, Malak Abdulaziz Almozaen, Rimas Sabih Alharbi

**Affiliations:** 1 Department of Preventive Dental Sciences, College of Dentistry, Taibah University, Al- Madinah Al-Munawwarah, 42353 Saudi Arabia; 2 College of Dentistry and Hospital, Taibah University, Al-Madinah Al-Munawwarah, 42353 Saudi Arabia

**Keywords:** Barrier, Research, Qualitative research, Dental education, Undergraduate dentistry

## Abstract

**Background:**

Research is an important part of dental education. While several quantitative studies have examined the barriers that undergraduate dental students face when conducting research, qualitative studies exploring the details and contexts of these barriers are rare. To bridge this gap, this research qualitatively explores the barriers faced by undergraduate dental students when conducting research.

**Methods:**

The present qualitative study employs what is known as the “performative knowledge strategy.” The 14 study participants were dental trainees included 10 undergraduate students and 4 interns who participated in research projects that were not part of their mandatory curriulum requirements. Diaries and semi-structured virtual (online) interviews were used to collect the data. Finally, using an inductive strategy, a thematic analytical approach was conducted to analyze the data.

**Results:**

Six key themes emerged from the data analysis. The themes of “inadequate knowledge,” “time constraints” and “lack of resources” confirm and align with the findings of previous studies, while the themes “misaligned schedules,” “delayed response” and “lack of orientation” represent new insights into the barriers to research faced by undergraduate dental students.

**Conclusion:**

This study identified six factors that posed substantial barriers to research by undergraduate dental students. Analysis of these barriers can lead to the design and implementation of strategies to enhance and improve research experience. Additional qualitative research in this area would help to reveal further details of the barriers to research, while additional quantitative studies are required to assess the frequency and trends of the newly revealed barriers.

**Clinical trial number:**

Not applicable.

**Supplementary Information:**

The online version contains supplementary material available at 10.1186/s12909-025-06790-y.

## Background

Undertaking meaningful research as an undergraduate student can improve students’ educational experience [1]. In particular, research helps to enhance students’ academic performance [2], develops their skills in their chosen field, helps them to decide what they wish to focus on as a postgraduate [3], and improves their critical thinking abilities [4]. In summary, by engaging in research as an undergraduate, students benefit from a more enriching educational experience that provides them with academic as well as professional skills that will help them in their careers [5]. For students who wish to continue to graduate study, an undergraduate research experience is vital to helping them secure places in these advanced courses [3] as research is an essential entry requirement for dental residencies, and is also required for many dental specialty board certifications, and other advanced professional degrees that dentists may pursue later in their careers [6]. The existing literature reveals that students are aware of the importance of research experience to their academic and professional development [7, 8]. Given the above, Adebisi [4] argues that the final-year research project reports, required by many dental schools internationally, are insufficient research experience for undergraduates, and recommends that students should engage in research experiences throughout their university studies.

Despite the importance of research to the dental education experience, only a few studies have explored the barriers that undergraduate dental students face when conducting research [8–11]. There is, however, a larger body of research that documents the research barriers experienced by health sciences students in general and other specific groups including medical students, medical and dental students, and dental students and dentists [12–21].

The literature has identified several barriers to research faced by students. A core barrier referenced in several studies is that students lack the time to engage in research [12, 14, 20]. Abdulrahman et al. [12] explained that this lack of time may be due to students’ demanding curricula. Study participants have also reported that the lack of mentors and supervisors to oversee students’ research activities is another major obstacle to conducting studies [8, 12, 20]. [12]. Additionally, it has been reported that students have little interest in conducting research [17] and are not motivated to do these types of investigations [16].

In addition to the above, several studies [12, 15, 20] identified a lack of related skills or knowledge as an important barrier to research by student populations. To illustrate this, it has been shown that students do not believe that either the teaching of research methodologies [9, 19] or the exposure to the required biostatistics programs, are adequate [9]. Another reported barrier is inadequate resources. Specifically, research reports indicated that students considered accessing journals and data to be a challenge [15, 18]. A further significant barrier to students conducting research is the lack of adequate laboratory facilities [16]. Lack of rewards or incentives have also been identified as a barrier to research by undergraduate students [20].

Important findings have been reported in these studies of health professions students’ research experiences, but three gaps exist in this literature [8–21]:

- First, there is little research into the actual research experiences of undergraduate dental students specifically.
- Second, the literature in this area rarely included detailed qualitative methodology, which is vital if we are to understand the details and contexts of student research obstacles.
- Third, previous studies of student research barriers were designed to elicit opinions and perceptions of what a barrier is and the significance of these barriers from students [8–11].

However, this is not the premise of the current study. No study has directly examined the reality of the barriers to research faced by undergraduate dental students as reflected in the practices of these students. What needs to be addressed instead, is to focus on how these barriers are embodied as actions in students’ research activities. Therefore, the purpose of this study was to explore the reality of the barriers faced by undergraduate dental students using a qualitative methodology and as manifested as a series of practices in their lives as students. This study thus contributes to the current literature on dental education and research and offers important findings that can inform improvements in undergraduate dental programs.

## Methods

### Study design

A qualitative research methodology was employed for data collection and analysis. This report is a component of a larger study, intended to establish a thorough understanding of undergraduate dental students’ research experiences.

### Study context

The study context for this research was the Western Region, the Kingdom of Saudi Arabia. Dental students from a single university were enlisted as study participants. All participants were dental undergraduates and dental interns in the process of completing their internships.

### Sampling strategies and participant recruitment

Two sampling strategies were used in this study: purposive and snowball sampling. As per the purposive sampling strategy, dental students and interns with research experience in a research project that did not form part of their mandatory coursework were selected for inclusion in this study. As data was being collected from this first group of participants, snowball sampling was used to recruit further research subjects. Specifically, snowball sampling involved asking the initial participants to suggest other potential participants who satisfied the inclusion criteria. The researchers then approached these individuals via WhatsApp. After explaining the study and inviting them to participate, arrangements for an interview was finalized for interested individuals. A topic guide was then sent to all participants before the interview to help them understand what types of questions they would be asked. No further participants were enlisted when data saturation was achieved, as described by Ritchie and Spencer [22].

### Data collection methods

This study employed two data collection methods: virtual semi-structured interviews or diaries for student participants who preferred to write their responses to questions rather being interviewed. The participants were given the option of either completing an interview or answering the questions in a diary format. The diaries contained the same open-ended questions asked in the virtual interviews. The interviews were mainly conducted in Arabic, but the participants were free to speak English if they wished. The participants who completed diaries received the diary entry prompts in both Arabic and English and could complete their responses in either language. When the participants were provided with the diaries, they were also asked to contact the research team if they had any questions about the diary prompts to ensure their answers included the required information.

It was often several days before the diaries returned their entries. The data collection process took place from 16 March 2024 to 7 April 2024.

### Data collection instruments and technologies

Zoom and Microsoft Teams were the platforms used for online interviews. The participants who completed a diary received the prompts for their diary entries via WhatsApp. Microsoft Word was used to write the diary entry prompts and the completed diaries were returned to the researchers by email. The topic guide or open-ended questions created for the interviews was informed by the literature as well as the academic work and research experiences of the investigators. The questions were designed to encourage the interviewees to speak openly about their experiences. The open-ended questions were:

1. Can you please share some details about your practical experiences conducting research?
2. Can you please share some stories or examples of the research that you have conducted or are currently undertaking?
3. Can you please share some stories about the barriers that you have faced when conducting or trying to conduct research?

To enrich the interview data and gather additional details, the interviewer asked participants to expand on their answers whenever appropriate. Moreover, the researchers used the information they collected in the first few interviews to further develop the topic guide and introduce new questions to enhance later interviews. An audio recorder was used to record the virtual interviews.

### Data processing

The researchers transcribed the interview audio recordings, while the diaries were already in written form and were thus ready for analysis. Security protocols and data management processes were used to protect the participants’ confidentiality. These protocols and processes involved anonymizing the interview transcripts and diary entries and preventing unauthorized people from viewing any of the collected data.

### Data analysis

The researchers analyzed the data following the steps for thematic analysis laid out by Braun and Clarke [23–25]. While the first author was responsible for the majority of the data analysis, the entire research team supported the analysis from beginning to end. Specifically, the first author (MMS) conducted one step of the analysis and upon completion, and then met with the rest of the research team to review and discuss their progress, with this process repeated for each subsequent step of the thematic analysis. Importantly, MMS used the feedback received from the research team to improve the analysis.

The data analysis process began with a detailed review of the first interviews and diaries. The researchers then familiarized themselves with the information gathered before creating the initial codes for the data. Next, phrases with similar codes were grouped to help the researchers identify potential themes. These potential themes were then reviewed and named. The researchers used a paper-based approach in this study and thus no computer software was used to analyze the data.

According to the definitions provided by Braun and Clarke [23], the themes identified in the present work were considered semantic as they reflected the direct meanings of the data. Additionally, this analysis also adopted a realist thematical analytical approach in that the researchers did not view the responses of the participants as the result of socio-cultural factors but as reflective of their authentic experiences. Additionally, inductive thematic analysis was employed in this study as themes were taken from the collected data and not pre-prepared based on existing theories. Finally, in line with Braun and Clarke [23] and Nowell et al. [26], the number of times a theme appeared in the research data was not considered to reflect how important that theme was.

### Knowledge position

In the present work, knowledge is understood largely through the lens of what Mol [27, 28] calls “performative knowledge.” Performative knowledge is not interested in people’s opinions and feelings; instead it focuses on what people actually do [27]. Importantly, the performative knowledge approach applied in this study was not designed to collect data on what the participants perceive as barriers to research and what they think about these barriers. Instead, this approach was used to understand how the barriers to conducting for research undergraduate dental students are reflected and embodied in their research activities as a series of practices. The performative knowledge approach adopted in this study is reflected in the questions asked in the interviews, for example, “Can you please share some stories about the barriers that you have faced when conducting or trying to conduct research?,” as they are concerned with the participants’ real-world actions. To reinforce this knowledge position, when participants expressed a perception, feeling, or opinion, this data was excluded from the data analysis.

### Strategies to enhance the trustworthiness and credibility of the data analysis

Member-checking and triangulation were used to confirm the trustworthiness and credibility of the data analysis. In this study, member-checking involved researchers asking participants to repeat an answer during the interviews where any clarification was needed to guarantee that the researcher fully understood the answer given. This technique ensured the researchers’ interpretations of the data and thus the accuracy of the study findings. To achieve triangulation, both interviews and diaries were used as the sources of the data collected.

### Ethical considerations

The study participants’ privacy and rights were safeguarded throughout the research process. The confidentiality and anonymity of the participants were protected and their data remained private at all times.

Finally, where relevant, this study report follows O’Brien’s [29] guidelines for the reporting of qualitative studies.

## Results

A total of 14 participants were recruited for this study. The group consisted of six men and eight women aged from 21 to 25 years old. Ten of the participants were in their fourth, fifth, or sixth year of dental school and four were interns. Table 1 contains a summary of the additional characteristics of the participants who completed interviews (8) or wrote responses to questions in diaries (6). Six core themes were generated by the analysis of the data collected in the diary entries and virtual semi-structured interviews.

**Table 1:** Study participant characteristics

Characteristic	Interviewed (*n* = 8)	Diary Entries (*n* = 6)
Gender		
Male	1	5
Female	7	1
**Age range (Years)**	21–25	21–25
**Year**		
4th Year	2	0
5th Year	0	6
6th year	2	0
Intern	4	0

### Inadequate knowledge

The theme of “inadequate knowledge” was identified from the data as a core practical barrier to students conducting research in undergraduate dental programs. The data shows that dental students face some practical obstacles to engaging in research due to their lack of knowledge regarding the various elements of the research process. This lack of knowledge and skills affects the entire lifecycle of a research project. The data also reveals that dental students lack research experience and thus have some theoretical understanding but little practical knowledge. The following quotations reflect this research barrier:

“We faced a problem in that we (students) were unable to search for existing literature in one of the search engines. We didn’t know how; we needed a supervisor to show us how to do it step by step…We had studied this search engine before (as part of the curriculum), but when it came to doing it in practice, we couldn’t…We were unable to find papers and references related to our topic.” (P 1).

“In one of the research projects that I participated in, the supervisor asked me to write the discussion. At that time, I had never written a discussion. I didn’t know how to do it; it was my first time. I told the doctor that I had never written a discussion section before, this means I didn’t know how, and she told me to look at previous papers to see how it’s done.” (P 6).

### Time constraints

“Time constraints” is a further theme generated by the data and identified as another important practical barrier to conducting research for undergraduate dental students. The data analysis indicates that undergraduate dental students face time shortages when carrying out research activities. Demanding course requirements and the need to study for classes and exams are a practical hindrance to students seeking to progress in their research. The following responses illustrate this theme:

“The doctor required us (students) to write a summary of our topic before the background. Time was a problem as during the study days we had exams, but we had to do it if we wanted to participate in the upcoming research day. We were unable to balance the two (studying and conducting research), so we postponed the work to the holidays. After the holidays, we started to do the background. It took time; we finished university; we went back home; we worked all day…sometimes we were unable to complete work on the project. We had exams.” (P 1).

“The supervisor told me to write up the results of our research. At that time, we were waiting for certain data so we could start writing, but the data was ready just before several days of exams. I was unable to write up the results, so we rescheduled the write-up.” (P 7).

### Lack of resources

According to the data, a lack of resources, such as necessary tools and technology, is a further practical barrier to research facing undergraduate dental students. This research barrier is reflected in the following quotes:

“When gathering and searching for evidence, we found great articles related to our topic, but we weren’t able to access them. We needed to pay for access.” (P 9).

“I was looking for certain data analysis software. I didn’t have it, and I had been informed by the supervisor to go to a certain place and get it. I contacted them but they didn’t have it. I was searching for it until another doctor helped me.” (P 12).

“I didn’t have a laptop, and they (the supervisors) required us to use software for citations and I didn’t have a laptop to download the software. I had to take my laptop from home, but I was still not able to use that software. I organized my references manually.” (P 3).

### Misaligned schedules

This theme highlights another significant barrier faced by dental students when conducting research, namely, “misaligned schedules.” Undergraduate dental students often find that their academic schedules conflict with other timelines and this hinders their research progress. Their schedules were misaligned with the timeline required to complete the various steps in the research process. Additionally, students’ schedules were misaligned with those of the supervisors who provided guidance and support for their research. The following responses illustrate this research barrier:

“I participated in research that required me to meet patients to take data from them. The problem was that let’s say half of the sample was available in the clinic at a time that conflicted with my schedule. I was unable to meet with them as I had lectures at that time. At the beginning, I started to collect data… (and) I had to miss some lectures, or sometimes I had to leave my (fifth-year) clinical sessions to go to the clinical sessions for sixth-year students (to meet with patients). That’s why I faced some difficulties at the beginning.” (P 13).

“We started to progress in our project with our supervisor, but we had a problem with our schedule and his schedule as we were both only free one day a week. We finished the required tasks on Sunday, but we had to wait until Thursday when our schedules aligned to get advice from him and explain our ideas to him.” (P 8).

### Delayed response

Another core barrier that emerged from the data analysis is “delayed response,” that is, the delayed response of supervisors to dental students conducting research. The data showed that when the participants reached a point in their research where they needed to report to their supervisors to get advice on how to proceed, the supervisors were typically unresponsive for some time. The following quotations explain this situation in detail:

“The communication between us (the students) and him (the supervisor) was through WhatsApp. We sent the work to him but he used to not respond to us.” (P 7).

“In one of the research projects that I participated in, we sent the document to the supervisor (to check and advise us what to do next) and for weeks, we received no response at all. Then, after that, they responded to tell us what we had to do for the next step and that we needed to finish it in two days.” (P 10).

“I contacted a supervisor to participate in research…(and) I started to work with her. At the beginning… I was contacting her but was getting no response. I called her but she didn’t answer. I had to check her schedule to see when she was in the clinic to meet her face-to-face.” (P 6).

### Lack of orientation

The final core barrier to research for undergraduate dental students that emerged from the data is “lack of orientation.” According to the data, students are sometimes unable to progress in their research as they do not know what the next research goals or steps are.

The following quotations describe this situation:

“We were working on a certain research project. We were sharing ideas but we didn’t agree on one of them. At the same time, the next steps were not clear to us. Our supervisors did not clarify to us what the next steps were. Some ideas were thrown around and that was it. Time was passing.” (P 11).

“One supervisor contacted me to conduct a research project and she sent me the proposal to fill in and informed me in general what we were going to do. But, when I started to work, I didn’t know how to do it, what to do, who I should send this (proposal) to after I finished; I had to learn by myself and figure it out.” (P 3).

## Discussion

To the best of our knowledge, this is the first study to qualitatively explore the reality of the barriers to conducting research faced by undergraduate dental students as manifested by the practices of these students. The study identifies six themes: inadequate knowledge, time constraints, lack of resources, misaligned schedules, delayed response, and lack of orientation.

Research focused specifically on the barriers to research faced by undergraduate dental students is limited [8–11] and all of the existing studies on this topic are quantitative excluding one [8] in which data was collected through the use of closed and open-ended questions. However, more literature on research barriers is available if we consider studies that target both dental students and dentists [12], only medical students [15, 17, 19, 21], both medical and dental students [, 14, 18, 20], or all students of the health sciences [13, 16].

Viewing the literature through this wider lens, several studies discuss the research barrier of a lack of necessary knowledge and skills [9, 12, 14, 20], with a considerable number of participants across these studies reporting their perception that this lack was hindering their research activities. For example, in one study [12], a significant amount of the study participants (38.76%) reported perceiving their lack of knowledge and skills as a barrier to research. Further research [14] looked specifically at the factors of a lack of knowledge, inadequate research skills, and inadequate statistical methods skills. In this study [14], many (38.5%) of the participants strongly agreed that a lack of knowledge was a barrier to their research activities, a majority (53.5%) strongly agreed that inadequate research skills were a barrier, and a considerable number (45%) strongly agreed that inadequate statistical methods skills were hindering their research progress.

From these studies [12, 14], it can be seen that a significant proportion of students consider a lack of knowledge and skills to be a barrier to research. Our study confirms these findings as inadequate knowledge was again found to be a barrier to research for the study participants. However, our study goes further to explore in detail how this barrier was reflected in the students’ practical research experiences. We found that a lack of knowledge and skills impacted various stages of the research process, such as sourcing existing literature and writing up the discussion section, and thus hindered the participants’ research progress.

Several studies also report time constraints as a barrier to research [8, 9, 12, 14, 20]. For example, 22% of the participants of one study [8] reported that time was a barrier to their research. The majority of respondents in another study [9] agreed with the statement that there is insufficient time when attending dental school to engage in research. In further research [12], 32.55% of the participants reported that they lacked sufficient time to dedicate to research because of their degrees’ heavy workloads. Our study supports these findings and again goes beyond what is reported in the existing literature by providing more context and details about this barrier. The theme of time constraints demonstrates the participants’ inability to find sufficient time to balance the demands of their studies with those of conducting research, leading them to miss lectures or postpone their research work until the holidays. This theme also reveals that the participants had to stop working on their research projects entirely when they had exams they needed to prepare for.

The lack of research resources, facilities and tools is another key barrier to research for students that is discussed widely in the existing literature [13, 16, 18, 20]. For example, it has been reported that students perceive difficulties in accessing data and journals [18, 20]. This finding is similar to our study, which found that students were unable to access certain articles as they needed to pay for access. In one study [20], 63% of the participants perceived a lack of facilities as a barrier to conducting research. Another [16] found that inadequate laboratory facilities were a major barrier to research for the students surveyed.

In addition to confirming the results of previous research, this study expands the existing literature on the barriers to research facing dental students in two main ways. First, this study reveals new barriers to research represented by the themes of misaligned schedules, delayed response, and lack of orientation. This research was able to uncover these new insights because of its qualitative design, which is sensitive enough to capture previously unidentified data. The additional context and details revealed by our study as compared with the limited findings of previous research can similarly be attributed to the qualitative nature of the study design and its ability to capture narrative data to uncover richer information. Importantly, these additional details can inform the design and implementation of interventions to improve and develop dental students’ research experiences and dental education in general.

Second, from a knowledge position perspective, our study stands out from the existing literature. Previous studies in this area adopt the same epistemological position; that is, they seek to understand the barriers to research from the point of view of the participants by asking them whether they “believe,” “perceive,” or “think” that something is a barrier. This can be described as the “perception-based approach.” For example, a lack of knowledge and skills is identified as a barrier to research in the existing literature because the study participants perceived that it was a barrier. While this approach to obtaining knowledge by capturing participants’ perceptions is valuable, it has certain limitations. To illustrate, an assessor could evaluate the participants’ knowledge and skill levels and find that they have sufficient knowledge but lack the confidence to apply it in practice. So, the findings reported by these studies on a lack of knowledge being a barrier to research could be inaccurate.

In contrast, our study applies the performative knowledge strategy described by Mol [27]. Instead of taking an indirect approach and asking participants about their perceptions of the reality under investigation (whether they consider inadequate knowledge to constitute a research barrier, for example), our approach directly examines the reality itself and how it is reflected in practice. Thus, we examine the actions performed by undergraduate dental students without reference to their opinions and feelings. The performative knowledge approach shows how, for example, inadequate knowledge was manifested in the participants’ actions and how they were unable to proceed with their research as they did not have the required knowledge (e.g. they had no experience writing a discussion section) to take the next step. This form of knowledge is more valid than others as it reflects the reality itself and is thus better able to determine whether the barrier being faced is, for example, a lack of confidence or a lack of knowledge. This performative knowledge approach has been implemented successfully before in dental education research [30].

### Research implications

The findings of this study have numerous implications. Regarding time constraints, it is recommended that undergraduate dental students be supported to develop strong time management skills. Moreover, formally integrating research into undergraduate dental curricula as a core learning activity will help to address the issue of time constraints because time for research work will be included in students’ study time. Additionally, including modules on research methods early in dental students’ studies can help them overcome the barrier of inadequate knowledge by equipping them with the fundamental skills and knowledge they need to conduct research. Regular research workshops exploring various elements of the research process would also help to resolve the issue of inadequate knowledge, as would assigning postgraduates or staff as mentors to undergraduate dental students to help them bridge any gaps in their research knowledge and skills.

Assigning postgraduates to mentor undergraduates will help them, in particular, with key skills such as statistical analysis and report writing. In terms of the barrier of a lack of resources, educational institutions must support their students by giving them the resources they need to efficiently complete their studies. The barriers of misaligned schedules and supervisors’ delayed response to students can be addressed by universities requiring supervisors to commit certain office hours to helping students with their research projects. These hours should be clearly communicated to students so they can fully benefit from this time. Finally, the barrier of lack of orientation can be resolved by requiring supervisors to meet with research students regularly to give them clear instructions and advice about the different phases of research and their objectives.

### Study limitations

This research has three key limitations. First, it collected data through virtual interviews and diaries only, with no data collected through observations. The use of data collected through observations could have strengthened the study. Second, this study focused on the practices and actions of the participants that reflected barriers to their research activities. It did not capture the participants’ needs, concerns, or thoughts regarding what they felt could help them to overcome these barriers. Third, the research barriers reported in this paper concern voluntary research projects that were not included in the students’ mandatory academic programs. Therefore, the study findings are not widely generalizable as some of these barriers would not apply to projects that formed part of the student’s mandatory program as such research would typically be well-supported by universities.

### Future research

We recommend two future research directions. First, given that qualitative studies into the barriers to research among undergraduate dental students are rare, further qualitative research is required to uncover new contexts, details, and meanings of research barriers. Second, quantitative studies focused on dental students are still needed to assess the new themes generated by this study, namely, misaligned schedules, delayed response, and lack of orientation. These studies must use adapted and refined quantitative tools (e.g. questionnaires that address these themes) to determine the themes’ frequency and significance for dental students.

## Conclusions

This study confirms the findings of previous research that inadequate knowledge, time constraints, and lack of resources often are barriers to dental student research. However, by implementing a qualitative methodology, this study uncovered additional barriers not identified in the existing literature. These additional barriers include misaligned schedules, delayed response and lack of orientation which describes a situation where students are unaware of the goals and procedural steps in the research process. To address these barriers, universities, colleges, and other educational institutions must implement various targeted interventions that support and facilitate the research activities of undergraduate dental students. In this way, the educational experiences of dental students and health sciences students in general can be meaningfully enhanced.

## Electronic supplementary material

Below is the link to the electronic supplementary material.

Supplementary Material 1

## Data Availability

The dataset supporting the conclusions of this article is included within the article and its additional files.
