# Supplementary material for: Barriers faced by undergraduate dental students when conducting research: a qualitative study
Source: BMC Med Educ. 2025 Feb 6;25:191. doi: 10.1186/s12909-025-06790-y (PMC11804027; doi:10.1186/s12909-025-06790-y)
Supplement: Supplementary file 1 — Supplementary Material 1 [file 12909_2025_6790_MOESM1_ESM.docx]

**Topic guide**

The topic guide for this study included three main questions, these are as follows:

1. Can you please share some details about your practical experiences conducting research?
2. Can you please share some stories or examples of the research that you have conducted or are currently undertaking?
3. Can you please share some stories about the barriers that you have faced regarding your research experience?
